# Mechanisms of vascularization in murine models of primary and metastatic tumor growth

**DOI:** 10.1186/s40880-016-0083-5

**Published:** 2016-02-12

**Authors:** Edina Bugyik, Ferenc Renyi-Vamos, Vanessza Szabo, Katalin Dezso, Nora Ecker, Andras Rokusz, Peter Nagy, Balazs Dome, Sandor Paku

**Affiliations:** 1st Department of Pathology and Experimental Cancer Research, Semmelweis University, Üllői út 26, Budapest, 1085 Hungary; Department of Thoracic Surgery, Semmelweis University-National Institute of Oncology, Budapest, 1122 Hungary; Hungarian Academy of Sciences Postdoctoral Research Programme, Budapest, 1051 Hungary; Department of Thoracic Surgery, Medical University of Vienna, 1090 Vienna, Austria; Department of Biomedical Imaging and Image-guided Therapy, Medical University of Vienna, 1090 Vienna, Austria; Tumor Progression Research Group, Joint Research Organization of the Hungarian Academy of Sciences and Semmelweis University, Budapest, Hungary

**Keywords:** Vascularization, Primary tumor, Metastasis, Angiogenesis

## Abstract

Directed capillary ingrowth has long been considered synonymous with tumor vascularization. However, the vasculature of primary tumors and metastases is not necessarily formed by endothelial cell sprouting; instead, malignant tumors can acquire blood vessels via alternative vascularization mechanisms, such as intussusceptive microvascular growth, vessel co-option, and glomeruloid angiogenesis. Importantly, in response to anti-angiogenic therapies, malignant tumors can switch from one vascularization mechanism to another. In this article, we briefly review the biological features of these mechanisms and discuss on their significance in medical oncology.

## Background

Angiogenesis refers to the proliferation of vascular cells (endothelial cells and pericytes) and the increase of vessel density. It has a pivotal role in the nutrition of tumors. In the 1970s, Folkman et al. [1] found that, without angiogenesis, solid tumors cannot grow beyond the limits of diffusion (1 mm^3^). Above this size, the so-called angiogenic switch occurs; currently, anti-angiogenic therapies are based on this assumption. In this article, we review and compare our data on the vascularization mechanisms of primary and metastatic tumors with the findings of other groups. We also discuss the potential reasons for failure of anti-angiogenic therapies [2, 3].

### Angiogenic processes during vascularization of tumors

The two basic forms of angiogenesis are endothelial sprouting [1] and intussusceptive angiogenesis [4]. Both processes require connective tissue and participate in the formation of the tumor vasculature. Many primary tumors (e.g., melanoma, colon cancer, and breast cancer) are surrounded by provisional connective tissue that develops because of the vascular permeability-enhancing effect of vascular endothelial growth factor (VEGF). This connective tissue contains fibronectin/fibrin and collagen, which enables the continuous development of new capillaries by sprouting or intussusceptive angiogenesis [5].

Two models of sprouting angiogenesis have been described. According to the first model, interendothelial contacts of the venules closest to the tumors become weakened [6]. This phenomenon is caused by angiogenic factors released by tumor cells. After local degradation of basement membrane, non-polarized endothelial cells migrate into the connective tissue. This is followed by the formation of the lumen, synthesis of the new basement membrane, and the appearance of pericytes around the newly formed capillaries. The disadvantage of this model is the inability to identify the stimulus required for lumen formation.

The second model suggests that interendothelial contacts remain intact, and after the degradation of the basement membrane, polarized endothelial cells migrate into the connective tissue [7]. During this process, parallel migration of polarized endothelial cells enables the prompt formation of a split-like lumen that is continuous with the lumen of the mother vessel. The basement membrane is continuously deposited by the endothelial cells. Only the very tip of the growing capillary bud is free of basement membrane and is in connection with the connective tissue collagen. Migrating along the basement membrane deposited by the endothelial cells, proliferating pericytes of the mother vessel appear later around the immature capillaries.

Intussusceptive angiogenesis (i.e., “vessel division”) is another mode to increase vessel density. This process enhances the complexity and density of the vessel network, thus providing additional surface for further vessel sprouting [4]. Intussusceptive angiogenesis increases vessel density faster than sprouting angiogenesis and plays a pivotal role in the formation of the developing lung vasculature [4]. There are two different modes of intussusceptive angiogenesis. According to the first model, endothelial cells of the opposite vessel walls establish contact because of the pressure generated by connective tissue cells or pericytes; this is followed by the reorganization of intercellular junctions leading to the perforation of the endothelial bilayer. Collagen-producing cells enter the perforation in the vessel lumen. These cells and the synthesized collagen build up the connective tissue pillar in the lumen of the vessel (1–5 µm in diameter), which is a characteristic of intussusceptive angiogenesis. Further growth of the pillar results in the complete division of the lumen [4].

The formation of intraluminal pillars may also occur by another mechanism, the first step of which is the formation of endothelial bridges in the lumen. Here, degradation of the basement membrane takes place, and bridging endothelial cells attach to connective tissue collagen bundles and transport them through the lumen. The collagen bundles are covered by endothelial cells; thus, initially, the nascent pillars are built up by two endothelial cells and a single collagen bundle. Fibroblasts migrate into this pillar and synthesize additional connective tissue, which results in pillar maturation [8]. Of note, similar to sprouting angiogenesis (model two; see above), the polarity of endothelial cells does not change in either case. Accordingly, this type of pillar formation can be called inverse sprouting since the connective tissue (surrounded by the endothelium) is located in the vessel lumen. In contrast, during sprouting angiogenesis, the lumen is located in the connective tissue.

Numerous schematic depictions in various studies suggested that, during vascularization, the nearby vessels grow into the tumor [1, 9, 10]. However, in 1987, Thompson et al. [11] found that mouse tumors grow by incorporating the host tissue microvasculature. A subsequent human study by Pezzella et al. [12] in 1997 confirmed the phenomenon of vessel incorporation in “non-angiogenic” primary non-small cell lung cancers. Subsequently, our research group proved in experimental and human melanomas that vessels that develop by sprouting angiogenesis in the connective tissue surrounding the tumors become incorporated. Because of the growth of the tumor, the vessel density of the incorporated vascular network decreases (becomes diluted) [13]. This phenomenon occurs because of the lack of branching activity of the intratumoral vessels. The low proliferation of endothelial cells solely supports the dilatation of the tumoral vessels [13]. An insufficient amount of collagen-containing connective tissue within the tumors results in the termination of sprouting angiogenesis. In both human and experimental melanomas, the capillary network is arranged in parallel with the surface of the tumor. This phenomenon itself supports the notion that melanomas do not acquire their vasculature by vessel ingrowth [13].

### Vascularization of metastases

In clinical oncology, treatment of metastases is a great challenge. Angiogenesis is a possible target for the treatment of tumors. Since the most frequent sites for metastasis formation (the brain, liver, and lung) contain dense capillary networks and a relatively small amount of connective tissue, it is likely that vascularization of metastases develops differently from primary tumors.

#### The brain

**Fig. 1:**
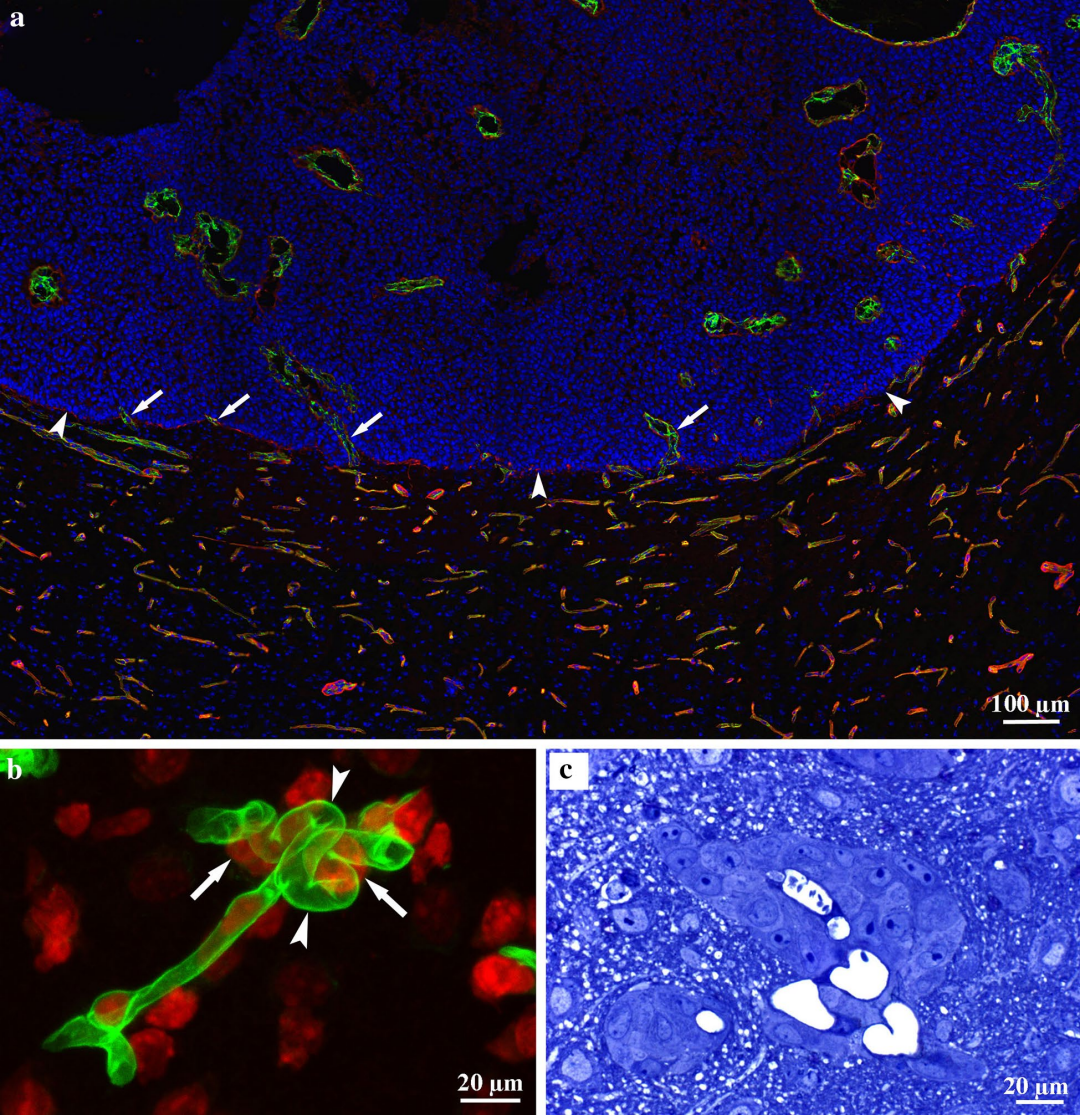
Vessel incorporation and glomeruloid body formation in the brain. **a** Frozen section of an experimental brain metastasis of the C38 colorectal carcinoma cell line. The section is stained for CD31 (vessels, *green*), laminin (basement membrane, *red*), and 4′,6-diamidino-2-phenylindole (DAPI; cell nuclei, *blue*). The DAPI staining shows that the tumor is located in the *upper* part of the picture. *Arrowheads* show the tumorparenchyma interface. The tumor mass “flows” around the vessels, thereby incorporating them (*arrows*). Vessel density is significantly lower and vessel diameter is larger in the intratumoral region than in the peritumoral tissue. *Scale bar* 100 µm. **b** Glomeruloid body formation after injection of tumor cells into the carotid artery. Simple loops (*arrowheads*) of the capillary (laminin, *green*) that develop in the vicinity of tumor cells (*arrows*) can be observed. Cell nuclei are stained by propidium iodide (*red*). *Scale bar* 20 µm. **c** Semi-thin cross section stained by toluidine-*blue* represents a glomeruloid body formed in a colony of human melanoma cells. There are several capillary lumens within the small tumor cell group. *Scale bar* 20 µm

It is widely accepted that angiogenesis occurs in primary and metastatic brain tumors and in other pathologic disorders [14]. It has been reported that vascularization of metastases can occur by the incorporation of brain capillaries [15]. We analyzed the vascularization of experimental brain metastases in detail using five different tumor cell lines. Tumor cells were inoculated directly into the brain parenchyma to enable tumors to reach the size at which angiogenic switch is thought to occur (1 mm^3^). We found no new vessel formation in the close vicinity of tumors. Vessel density and bromodeoxyuridine (BrdU) labeling index of vascular cells did not change significantly. Tumors became vascularized exclusively by the incorporation of host vessels (Fig. 1a). During incorporation, the tumor mass “flowed” around the vessels while tumor cells detached astrocytes from their surfaces. The growth pattern of the metastases is associated with the number and size of incorporated vessels. Tumors with infiltrative growth incorporated more vessels than expansively growing tumors (pushing type). We also found a negative correlation between the intratumoral vessel density and BrdU labeling index of vascular cells. Therefore, tumors with low levels of vessel incorporation may enhance blood supply by increasing vessel surface through vessel dilatation [16].

Glomeruloid bodies (GBs) are characteristics mainly of primary brain tumors, but they appear in brain metastases and in other tumors as well [17]. GBs acquired their name because of their superficial resemblance to renal glomeruli. Until recently, the mechanism of GB formation was largely unknown. Stiver et al. [18] performed a detailed analysis of GBs that were developed in a tumor-free environment using a VEGF-coding vector injected into brain tissue. The first step in the process was the appearance of dilated mother vessels. In the vessel wall, pericytes and endothelial cells started to proliferate and formed numerous lumens within the lumen of the mother vessel. Next, these structures differentiated into distinct daughter vessels through apoptosis of endothelial cells. Our group described another much simpler process in brain micrometastases [19]. According to this model, after injection of tumor cells into the carotid artery, tumor cells extravasate and adhere to the external surface of the basement membrane of capillaries. Following this, even a single cell is able to produce simple loops on capillaries (Fig. 1b), presumably because of the forces exerted by the actin cytoskeleton of tumor cells attached tightly to the surface of the basement membrane. Later, the force exerted by the proliferating and migrating tumor cells results in the appearance of more complex GBs as tumor cells pull new vessel segments into the tumor nests (Fig. 1c). This process leads to the thinning and eventual rupturing of capillary segments located between tumor nests. Proliferation of endothelial cells is only slightly elevated during this process; therefore, the increase of the vessel density is simply the consequence of the remodeling of the existing vasculature in the brain tissue.

#### The liver

Vermeulen et al. [20] described liver metastases with different growth patterns. The first type is the so-called “replacement” growth pattern where the liver structure is preserved and the liver trabeculae are replaced by tumor cells. In the second important growth pattern (“pushing”), the liver structure is distorted, liver cells on the surface of metastases are compressed, and connective tissue is deposited around the tumor. Replacement growth is a characteristic of undifferentiated metastases, whereas pushing growth is a characteristic of differentiated metastases.

We examined experimental liver metastases that were produced by the injection of Lewis lung carcinoma cells into the spleen of C57Bl/6 mice. In this model, tumor cells reached the liver via the portal vein and formed colonies. Our studies on anaplastic Lewis lung carcinoma liver metastases showed that tumor cells migrated in the space of Disse during the invasion, at the tumor periphery, thereby detaching the endothelial cells from their basement membrane (structured basement membrane could not be observed in the sinusoids, but immunoelectron microscopic observations revealed several basement membrane elements between the microvilli of hepatocytes) [21]. Endothelial cells that get into the tumors start to proliferate and form functional vessels with wide lumen and vulnerable, convoluted walls (sinusoidal type metastases) [21]. We also observed another type of Lewis lung carcinoma metastasis that was located in portal spaces or in their close vicinity (portal-type metastases). These metastases are characterized by numerous small vessels that stained positively for basement membrane components. These vessels presumably evolved during sprouting angiogenesis within the connective tissue of portal spaces. Notably, the ratio of portal-type metastases was higher when tumor cells reached the liver via the arterial system. However, tumor cells entering the liver from the direction of the portal system produced more sinusoidal metastases. These results suggest that a proportion of the tumor cells delivered via the arterial route are more likely to be trapped in the capillaries of the peribiliary plexus [21].

**Fig. 2:**
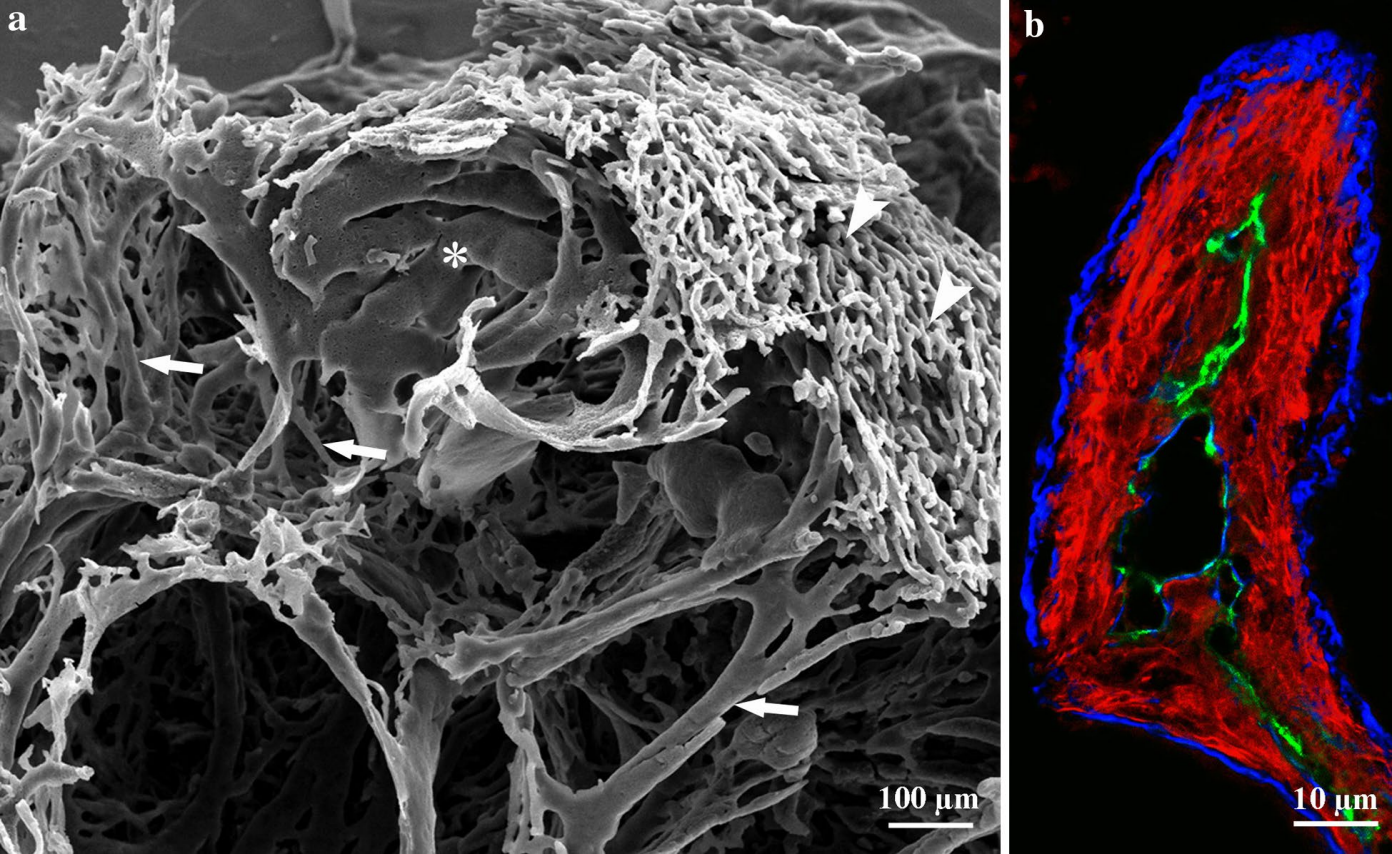
Vascularization and connective tissue column formation in C38 liver metastases. **a** Scanning electron microscopic micrograph of the vasculature of an experimental colorectal carcinoma metastasis in the liver after corrosion casting. On the *upper-right side* can normal structure of the sinusoidal system be observed (*arrowheads*). Sinusoidal lakes (*asterisk*) developed by fusion of the sinusoids can be observed close to the surface of the tumor. *Arrows* point at vessels supplying the metastasis. These structures correspond to the structure labeled by CD31 on Fig. 2b. *Scale bar* 100 µm. **b** The structure of (blood supply-providing) connective tissue columns in liver metastases. CD31-positive vessel (*green*) is located centrally and surrounded by smooth muscle actin (*red*)-expressing cells. The structure is surrounded by basement membrane (laminin, *blue*) deposited by tumor cells. Basement membrane (laminin, *blue*) of the central vessel can also be observed. Tumor cells (not stained) are located in the *black* areas. *Scale bar* 10 µm

Our analysis of the pushing-type metastases produced by using C38 colon carcinoma cells showed that even in the early stages of metastasis development, proliferation of myofibroblasts and accumulation of connective tissue occur in the close vicinity of the metastases [22]. In parallel, liver cells are displaced, which leads to the fusion of sinusoids on the surface of the liver metastases (Fig. 2a, b). Fused sinusoids become incorporated together with the connective tissue, and the inner part of the invagination is pinched off by the tumor tissue. As a result, connective tissue columns containing a central vessel will appear; these structures are able to provide blood supply for the tumor. Tumor cells surround these structures in a polarized manner and synthesize basement membrane onto the surface of the connective tissue (Fig. 2b).

Finally, it is noteworthy that in neither sinusoidal (replacement) nor pushing-type metastases does angiogenesis take place in the peritumoral liver parenchyma.

#### The lung

**Fig. 3:**
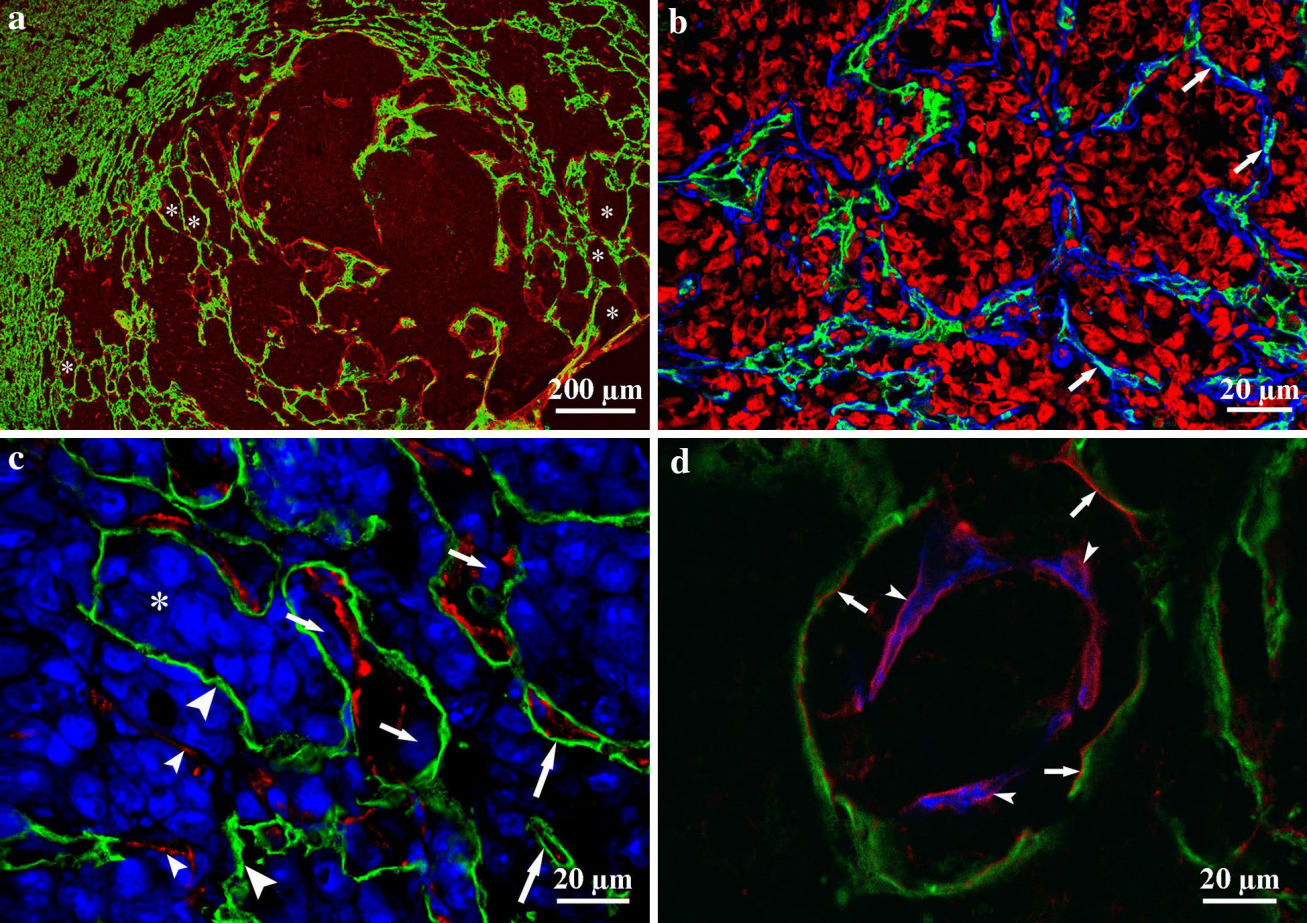
Vascularization of lung metastases. **a** Frozen section of a colorectal carcinoma metastasis in the lung. CD31 (vessels, *green*) and laminin (basement membrane, *red*) show that normal lung parenchyma is present (*left* *side*) next to the tumor tissue (*right* *side*). Tumor tissue is recognizable (*pale-red*) because of the laminin deposited by tumor cells. Alveoli are filled by tumor cells to different extents (*asterisks*). *Scale bar* 200 µm. **b** The peripheral region of a lung metastasis. Alveolar structure is preserved, and capillaries (CD31, *green*) are located within the alveolar walls (laminin, *blue*; *arrows*). The alveolar spaces are filled by tumor cells (propidium iodide, *red*). *Scale bar* 20 µm. **c** Lung metastasis of B16 melanoma cells. Tumor cells are stained by TOTO-3 (*blue*). Tumor cells (*small arrows*) can be observed between pneumocytes (podoplanin, *green*) and the vessel wall (CD31, *red*). The alveolar structure is mostly disintegrated; however, intact alveolar walls still can be observed (*large arrows*). *Asterisk* shows the original alveolar lumen filled by tumor cells. Denuded vessels (*small arrowheads*) and vessel-free pneumocytes (*large arrowheads*) are located within the tumor tissue. *Scale bar* 20 µm. **d** High-power confocal image of a lung metastasis. This *figure* shows that both the detached pneumocytes (podoplanin, *green*; *arrows*) and the vessel (CD31, *blue*; *arrowheads*) have their own basement membrane (laminin, *red*), and tumor cells are located between them. *Scale bar* 20 µm

In earlier studies, we found that the default growth type of experimental lung metastases is the “flow” of proliferating tumor cells from alveolus to alveolus [23] (Fig. 3a, b). This process leads to the formation of the alveolar pattern in primary human and metastatic lung tumors (non-angiogenic tumors), described previously by Pezzella et al. [12, 24]. Note that in the intact lung tissue around the metastases, proliferation of endothelial cells only slightly increases. This suggests that angiogenesis does not occur in this region. However, we found that vascularization of tumors is not completed by the occupation of the alveoli. Instead, it continues by different mechanisms in undifferentiated versus differentiated (desmoplastic) tumors [23].

Cells of undifferentiated tumors enter into the alveolar walls, and during their invasion/migration they detach pneumocytes from the surface of capillaries (Fig. 3c). As a result, tumor cells co-opt the capillaries that were formerly responsible for the gas exchange. Incorporated capillaries remain functional and provide blood supply for the tumor. Interestingly, tumor cells detach pneumocytes from the capillaries together with their basement membrane; thus, tumor cells actually migrate between the endothelial and epithelial basement membranes (Fig. 3d). Of note, the presence of basement membrane is not sufficient to support the survival of pneumocytes, which vanish by fragmentation within the tumor tissue.

Polarized tumor cells of well-differentiated C38 colon tumor do not migrate back into the alveolar walls from the alveolar space; instead, they induce a desmoplastic reaction in the alveolar wall. During this reaction, the fibroblasts present in the alveolar wall are transformed into smooth muscle actin (SMA)-expressing and connective tissue collagen-synthesizing myofibroblasts. Alveolar walls being incorporated into the tumor gradually widen, resulting in the development of connective tissue columns (centrally located microvessels embedded in connective tissue collagen and SMA-expressing activated fibroblasts surrounded by a basement membrane). The structure of these columns corresponds completely to the structure of connective tissue columns in the liver metastases of this same tumor (Fig. 2b). The accumulated connective tissue within the metastases and the significantly elevated proliferation index of intratumoral endothelial cells may indicate the initiation of angiogenesis within the columns.

### Blood supply of metastases

**Fig. 4:**
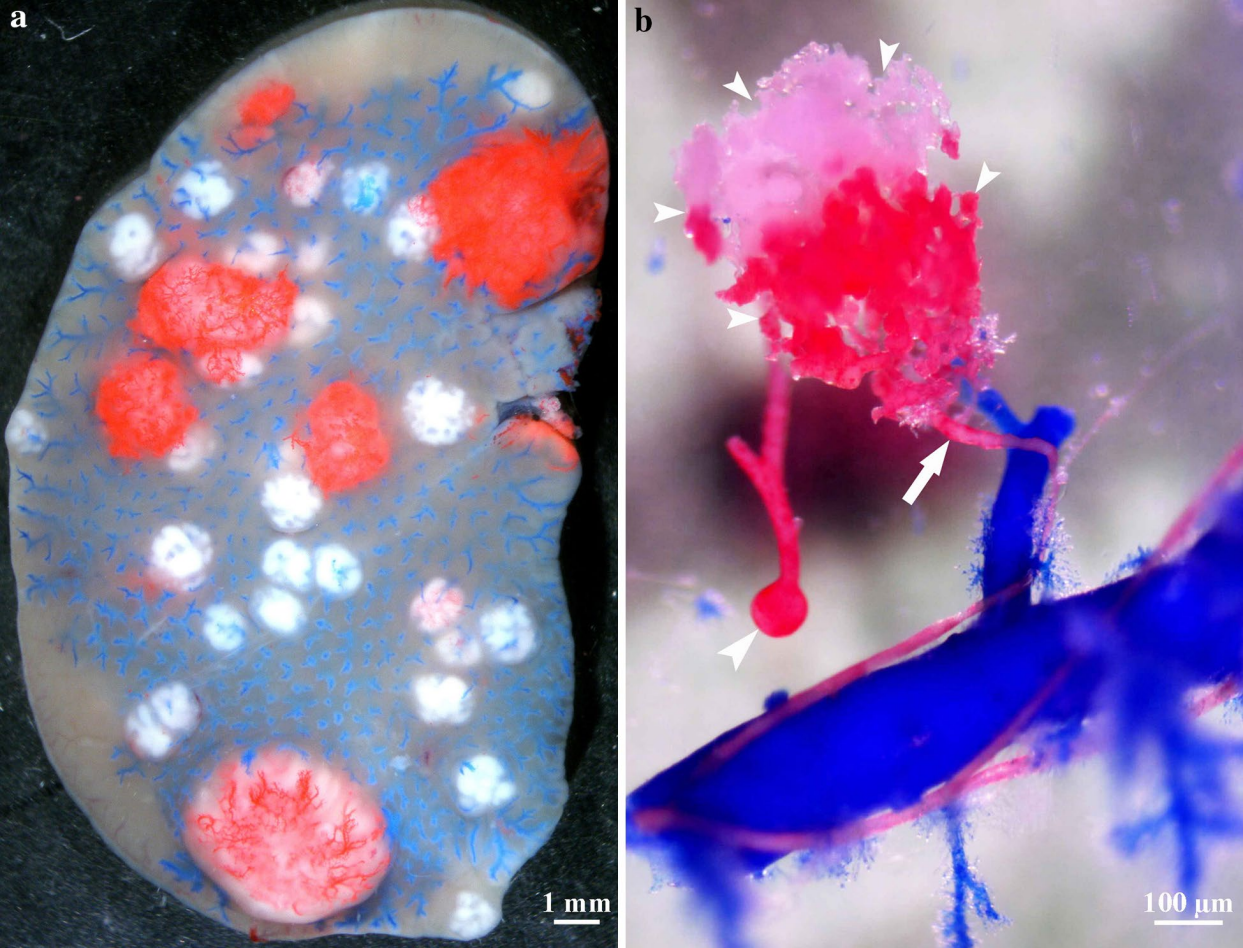
Blood supply of liver metastases. **a** Stereomicroscopic image of colorectal carcinoma liver metastases after two colored casting procedure. Blue resin was injected into the portal vein and red resin was injected into the arterial system. Smaller metastases supplied by the portal system appear white (since the portal system was filled up to the level of terminal portal venules, the resin does not enter the sinusoids and, consequently, it does not enter the small metastases). Red resin appears in metastases that are in direct connection with the arterial system. Note that all of these metastases are larger than those that appear white. *Scale bar* 1 mm. **b** An arterial metastasis after corrosion of liver tissue. The artery (*red*) runs next to the portal venule (*blue*) and enters the metastasis (*small arrowheads*) centrally (*arrow*). Moderate dilatation of the artery can be observed near the entry site (*arrow*). The red resin injected into the arterial system appears also in the central vein (*large arrowhead*). *Scale bar* 100 µm

In organs with dual blood supply (the liver and lung), the origin of the blood supply of metastases has been long debated. Most analyses have been done in the liver (including both animal and human samples). These experiments were performed by the injection of colored resin or India ink into the vasculature [25–27]. However, these studies neglected the relation of the arterial to the portal system and the anatomical differences between murine and human liver. Contrary to the human liver, mouse and rat livers have an extensive system of anastomoses between the arterial and portal system at every level of the vascular network. Moreover, rat lung has anastomoses between the bronchial and pulmonary arteries as well, which makes it difficult to determine the origin of blood supply. This problem can be solved by injecting casting solution into the portal vein or pulmonary artery up to the sinusoids or capillaries observed on the surface of the organs so the anastomoses between the two systems are blocked. Under these conditions, the resin injected into the arterial system will appear only in metastases that are directly connected to arterioles or arteries (Fig. 4a). We observed that liver metastases larger than 2.5 mm in diameter become arterially supplied [28]. However, in rat lung metastases, the bronchial artery is responsible for the blood supply of metastases larger than 5 mm only [23]. Supplying arteries are located mainly centrally, so it is likely that the blood flows from the center to the periphery (Fig. 4b). The difference regarding the tumor size in these two organs may be owing to the fact that, in the rat lung, bronchial arteries do not expand to the periphery of the lung, so the metastases growing in this organ reach the arterial system only when they get larger. The key step in the process of arterialization may be that low-pressure vessels (portal and central veins and pulmonary arteries and veins) are pushed aside by the tumor mass, whereas elements of the high-pressure arterial system become incorporated. Consequently, regarding delivery of chemotherapeutic agents into the metastases, the arterial system may play an important role in both organs.

## Conclusions

We conclude that the incorporation of both the preexisting host vasculature (which can be modified by the tumor) and the newly formed vessels plays an important role in the vascularization of tumors. The incorporation process is basically biomechanical in nature. We think that anti-angiogenic therapies should be given mainly to patients with primary tumors in which endothelial proliferation is present. The perimetastatic region cannot be targeted by anti-angiogenic therapies because angiogenesis does not occur in the adjacent host tissues of tumor metastases (possibly because of the lack of sufficient connective tissue). However, when sufficient connective tissue is synthesized in the more central part of the lesions in a later phase of metastasis development, intratumoral angiogenesis is possible. This requires the presence of connective tissue cells in the target organ that are able to transform into collagen-synthesizing myofibroblasts.
